# Spatial distribution of child pedestrian injuries along census tract boundaries: Implications for identifying area-based correlates

**DOI:** 10.1371/journal.pone.0179331

**Published:** 2017-06-14

**Authors:** Jacqueline W. Curtis

**Affiliations:** GIS Health & Hazards Lab, Department of Geography, Kent State University, Kent, Ohio, United States of America; Duke University, UNITED STATES

## Abstract

Census tracts are often used to investigate area-based correlates of a variety of health outcomes. This approach has been shown to be valuable in understanding the ways that health is shaped by place and to design appropriate interventions that account for community-level processes. Following this line of inquiry, it is common in the study of pedestrian injuries to aggregate the point level locations of these injuries to the census tracts in which they occur. Such aggregation enables investigation of the relationships between a range of socioeconomic variables and areas of notably high or low incidence. This study reports on the spatial distribution of child pedestrian injuries in a mid-sized U.S. city over a three-year period. Utilizing a combination of geospatial approaches, Near Analysis, Kernel Density Estimation, and Local Moran’s I, enables identification, visualization, and quantification of close proximity between incidents and tract boundaries. Specifically, results reveal that nearly half of the 100 incidents occur within roads that are also census tract boundaries. Results also uncover incidents that occur on tract boundaries, not merely near them. This geographic pattern raises the question of the utility of associating area-based census data from any one tract to the injuries occurring in these border zones. Furthermore, using a standard spatial join technique in a Geographic Information System (GIS), these points located on the border are counted as falling into census tracts on both sides of the boundary, which introduces uncertainty in any subsequent analysis. Therefore, two additional approaches of aggregating points to polygons were tested in this study. Results differ with each approach, but without any alert of such differences to the GIS user. This finding raises a fundamental concern about techniques through which points are aggregated to polygons in any study using point level incidents and their surrounding census tract socioeconomic data to understand health and place. This study concludes with a suggested protocol to test for this source of uncertainty in analysis and an approach that may remove it.

## Introduction

Many studies report a relationship between area-based socio-economic characteristics and injury [1–9] and the census tract is often the geographic unit in which these relationships are operationalized [10]. In particular, understanding of child pedestrian injuries has been advanced through such linkages, [11–15], which are more recently being facilitated through use of Geographic Information Systems (GIS) [16–22].

In such studies, it is standard to overlay a) points of incidents with b) census tracts. The points are then aggregated to the census tract in which they are located through a spatial join technique. Once the data are aggregated in this way, they can then be normalized by an appropriate denominator and analyzed with statistical approaches to show relationships between outcomes and their socioeconomic context.

This technique of aggregating points to the polygons in which they occur, spatial join, is generally performed using one of four options depending on the data being utilized and the objectives of data manipulation. Using ArcGIS terminology [23], these options are:

1) Points to Polygons: Each polygon is appended with a summary of the numeric attribute of the points that fall inside it, and a *count field of the points that fall inside it*. 2) Points to Polygons: Each polygon is appended with the attributes of the *point that is closest to its boundary*, and a distance field showing how close the point is. 3) Polygons to Points: Each point is appended with the attributes of the *polygon that it falls inside*. 4) Polygons to Points: Each point is appended with the attributes of the *polygon that is closest to it*. For numerous studies of health and place where outcomes or events are represented as points and the place is represented by tracts, Option 1 is the standard approach.

This is the most intuitive way to achieve a count of points in each polygon. In addition to enabling quantitative analysis with census variables, this data transformation has the benefits of improved visualization of spatial patterns and spatial confidentiality. For example, when looking at a point layer of many types of health outcomes in a city, it is difficult to make sense of their geographic distribution, depending on the data and scale of observation. One efficient way to quickly see areas of high versus low occurrence is to aggregate the points to some underlying administrative boundary and create a choropleth map. Furthermore, such a map has the added effect of masking the locations of individual outcomes, which is particularly important if the points represent residential locations. For these reasons of intuitiveness, visualization, and confidentiality, Option 1 is the standard approach for aggregating points to polygons. To date, there has been no reason to question its validity.

**Fig 1:**
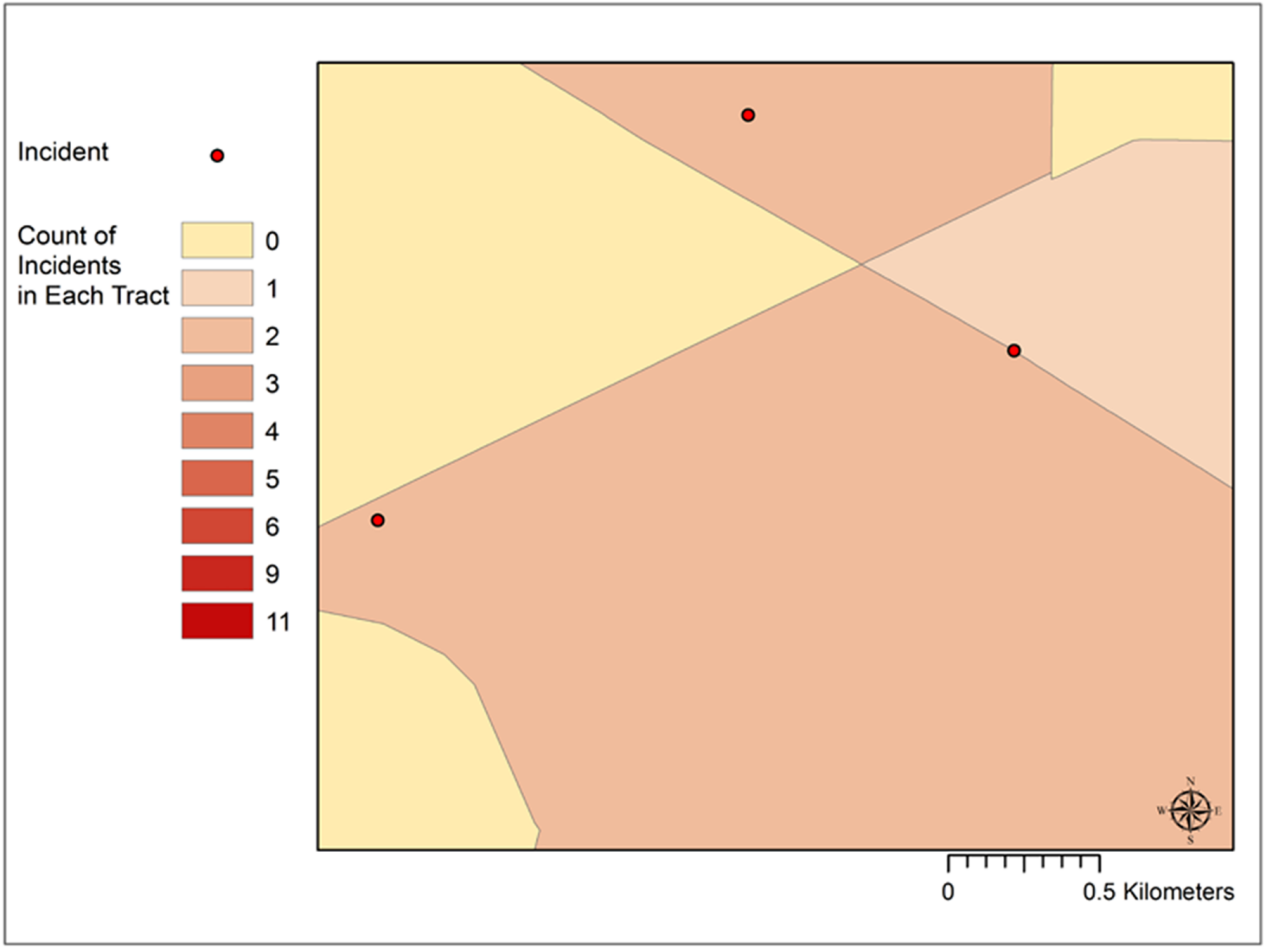
Example of close proximity of points to census tract boundaries.

This study was originally designed to include such a traditional analysis of area-based correlates of child pedestrian injury. However, in the process of initial mapping and aggregating point level incidents to their associated census tracts, two unexpected outcomes resulted. First, a striking geographic pattern was observed where many of these points were close to or even apparently on tract boundaries (Fig 1). Second, after aggregating these points to the census tracts in which they fall inside, the total number of incidents for the tracts was greater than the number of incident points. This observation suggests that the spatial join approach so widely used to aggregate points to polygons counts some points as falling inside more than one polygon. However, there is no indication to the user of this occurrence or where these multiple counts are located.

While such a coupling of incidents to tract boundaries is not expected in many health outcomes, pedestrian injuries are an exception. These events occur within or very near roads, and roads serve as one form of census tract boundary. If these points along the boundaries are being counted as falling inside all tracts that share this boundary, it is likely that these census tracts are being identified as having substantially high numbers of incidents based on what occurs at the outer fringes of these units.

Therefore, the aim of this study is to a) demonstrate the spatial distribution of child pedestrian injury incidents along census tract boundaries and then to b) test the implications of this specific geographic pattern in aggregating the point locations to the census tract in which they are located. The study concludes with a suggested protocol for avoiding the potential uncertainty that this pattern can introduce in examining area-based correlates and an approach that removes such uncertainty.

## Materials and methods

### Data

Police incident data on pedestrian-motor vehicle crashes in a mid-sized Ohio city occurring between January 1, 2013 and December 31, 2015 were acquired [S1 File]. In this 3-year period, 327 incidents were reported. Age of the pedestrian was reported for all but one incident (Table 1). All 100 incidents involving children had an accompanying x,y coordinate for the location of occurrence.

**Table 1:** Summary of the data by year.

YEAR	NUMBER OF REPORTED INCIDENTS	NUMBER OF INCIDENTS—VICTIM 18 YEARS OR YOUNGER	PERCENTAGE OF INCIDENTS– VICTIM 18 YEARS OR YOUNGER
2013	99	30	33%
2014	104	30	29%
2015	123	40	33%

The census tract boundary file created for the 2010 U.S. Census was acquired for the study area and both datasets (incidents and census tracts) were mapped in ArcGIS 10.4 [23]. Once in the GIS, the data were transformed from a geographic coordinate system in the North American Datum 1983 (GCS NAD 83) to Universal Transverse Mercator projection (UTM Zone 17 N) for analysis.

### Analysis

The spatial relationship between injury incidents and tract boundaries was first investigated using Near Analysis. First, census tracts were converted from polygons to lines using the Polygon to Line tool, a Geoprocessing Tool under Data Management > Features. Then, the distance between each incident and its nearest line (tract boundary) was calculated using Near Analysis under Analysis Tools > Proximity > Near. This approach created a new field in the incident attribute table labeled “NEAR_DIST”. As these calculations were conducted in UTM projection, results are reported in meters. To identify clusters in these data, a) Kernel Density Estimation (KDE) was performed and b) the “NEAR_DIST” values were used as weights when calculating spatial autocorrelation [24] with close distances being more heavily weighted. These approaches enable visualization of specific areas where incidents are located on or near census tract boundaries and would therefore raise concern about linkage with tract level socioeconomic variables in these places.

To further investigate how points are assigned to polygons based on location, especially in border regions, three relevant approaches were tested: Option 1 (standard approach), Option3, and Option 4. Option 2 is not tested as the output from this process does not result in the ability to count the number of points within each polygon.

These data and methods address the aims of this study to a) demonstrate the spatial distribution of child pedestrian injury incidents along census tract boundaries and then to b) test the implications of this specific geographic pattern in aggregating the point locations to the census tract in which they are located.

## Results

### Near analysis, hot spots, and spatial autocorrelation

**Fig 2:**
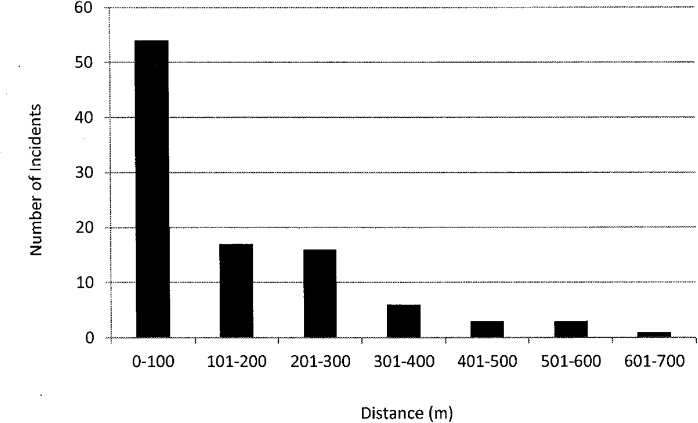
Results of near analysis: Number of incidents within specified distances from nearest census tract boundary.

Near analysis results in a range of incidents located from 0–633.48m from a census tract boundary, with an Interquartile Range (IQR) of 215.90m. Twelve of these points have a value of 0m. Fig 2 provides a summary of the distances of each incident to its nearest census tract boundary.

**Fig 3:**
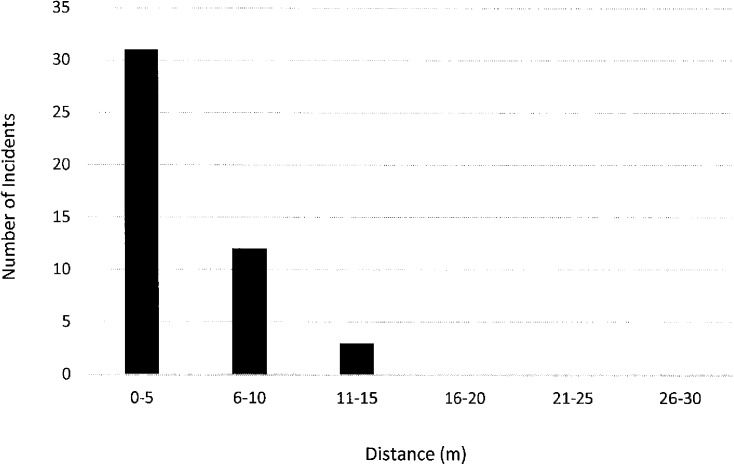
Results of near analysis: Number of incidents within 30m from nearest census tract boundary.

Furthermore, street widths were measured to identify a zone of census tract change, where the distance of the incident from the boundary is greater than zero, but still in close proximity. Based on these measurements, 30m was selected as a representative street zone of transition between tracts. Using this measure, all incidents were selected with a NEAR_DIST value of 30m or less. Forty-six incidents (46%) occurred within this zone of a census tract change and all of these points are 11m or closer to the boundary. Fig 3 illustrates the distribution of the points that are within a street width distance of the boundary.

These results identify a preponderance of incidents occurring just at the boundary from one census tract to another, and with some being located directly on the boundary itself. However, it is not enough to know that this pattern exists, but also where (e.g., is it present across the study area or only in a certain places), and what it portends for linking child pedestrian injury locations to their surrounding socioeconomic context. Therefore, KDE was used to visualize the presence of hot spots and then Local Moran’s I was employed test the significance in clustering of these border and near-border incidents.

**Fig 4:**
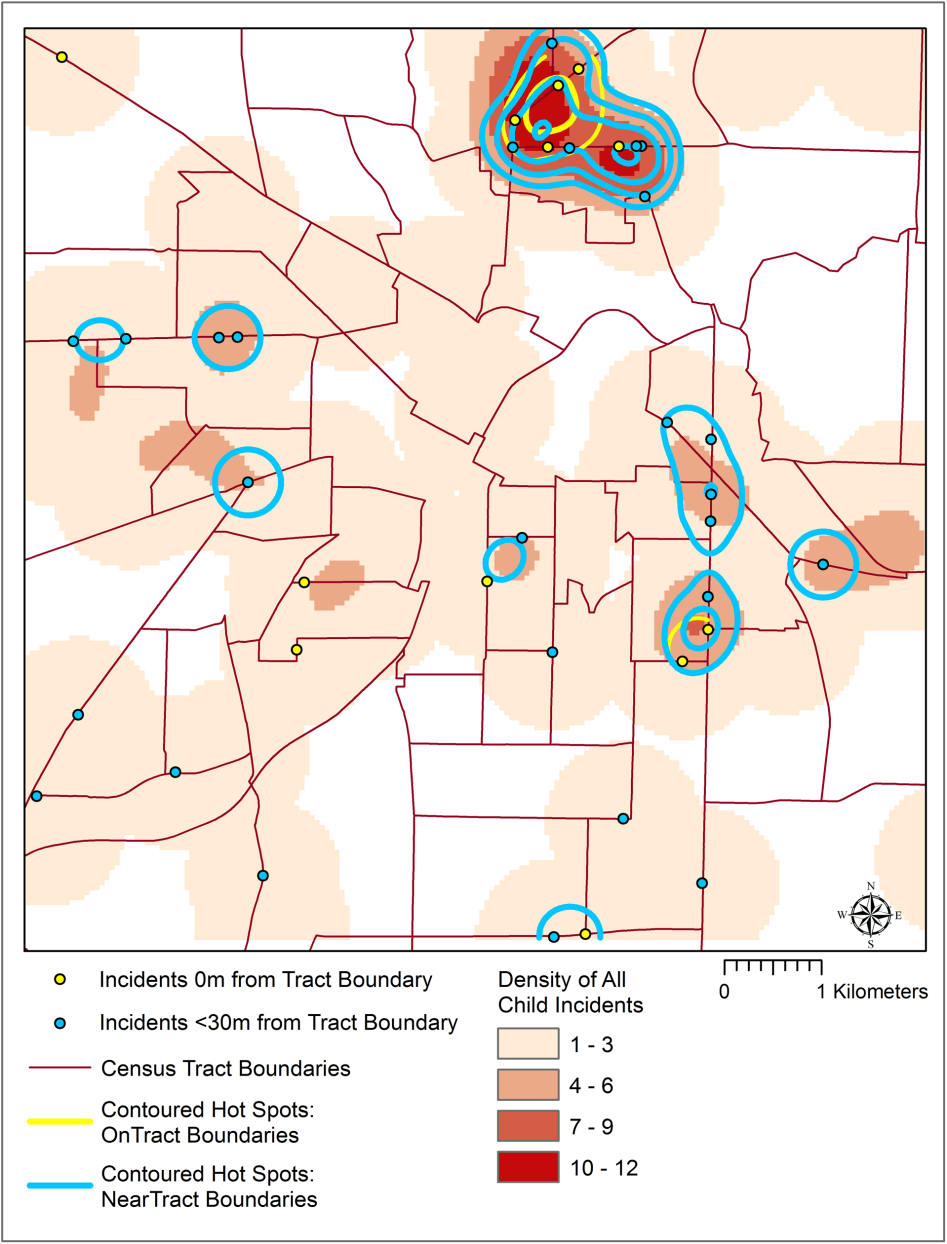
Spatial patterns of all incidents and those near or on a census tract boundary.

KDE (800m bandwidth) revealed that these intersecting or near-intersecting points are concentrated in the northeast quadrant of the study area (Fig 4), with smaller pockets distributed throughout the city.

In order to quantify the significance of this pattern of apparent clustering, Local Moran’s I was calculated using a fixed distance threshold of 3200m to ensure a minimum number of neighbors in the analysis and the distance calculated from the Near_Dist approach were used as weights. However, no areas met the criteria of statistical significance (*p<0*.*05*). Despite falling short of significance, visualizing their distribution is still valuable as it reveals a spatial pattern of the risk of introducing uncertainty in aggregation and the subsequent linkage with census tract socioeconomic data. These techniques demonstrate the spatial distribution of child pedestrian injury incidents along census tract boundaries which portends geographic variation in uncertainty within the study area for subsequent area-based analysis. The pattern exists and is concentrated more in some areas than in others, but how this pattern manifests in subsequent analysis requires further investigation through testing of a variety of common forms of aggregation.

### Spatial joins

The three relevant options that can produce a count of the number of points (incidents) in a polygon (census tract) each produced different results.

**Fig 5:**
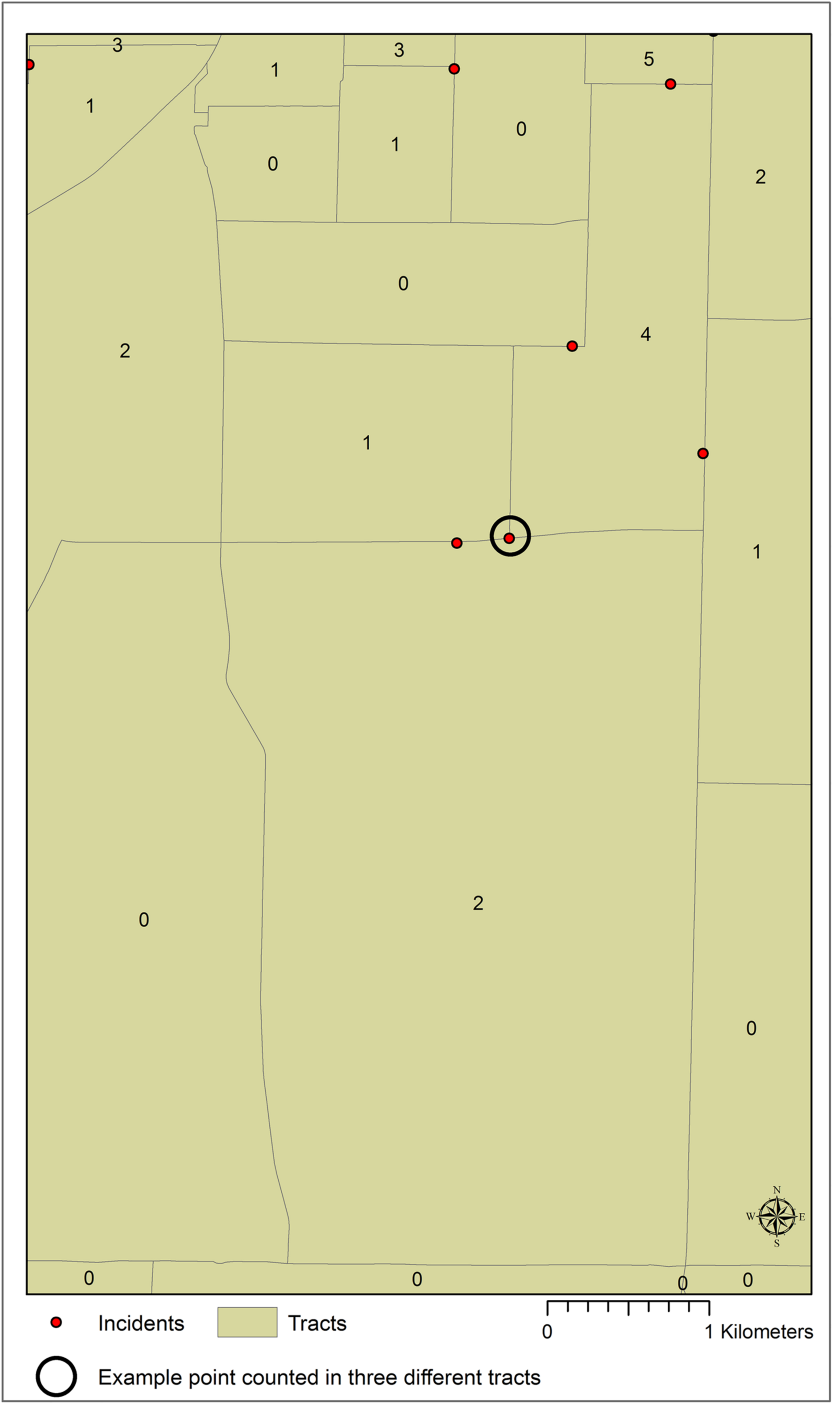
Example of one point counted in each of the three contiguous tracts.

Option 1: This approach resulted in 87 points being assigned to only one census tract, 11 assigned to two census tracts, and 2 assigned to three census tracts (Fig 5). The sum of all incidents is 115.

Option 3: This approach resulted in 13 of the points not be assigned to a census tract (where m = 0 through to 0.000016). The sum of all incidents is 100.

Option 4: This approach resulted in all points being assigned to only one census tract. The sum of all incidents is 100.

Comparing the results of Option 3 and Option 4, both yield a sum of 100 incidents which indicates an absence of assigning a point to more than one tract as occurred in Option 1. Examination of the census tracts reveals that of the 44 tracts with at least 1 incident occurring within its boundaries based on at least one of the spatial join approaches 17 (41%) demonstrated inconsistency in the number of incidents across the three approaches. Seventeen (100%) of the census tracts changed between Option 1 and Option 3 with a total difference of 28 incidents. Thirteen (76%) of the census tracts changed between the Option 1 and Option 4 with a total difference of 15 incidents. Seven (41%) of the census tracts changed between Option 3 and Option 4, with a difference of 13 incidents. Recall that Option 3 counted 13 incidents as NULL values and therefore these are not assigned to any census tract. Table 2displays census tracts in which the number of incidents attributed to it varied based on the spatial join approach. The grayed cells identify the anomalies.

**Table 2:** Number of incidents counted within census tracts based on different spatial join approaches.

TRACT_ID	OPTION_1	OPTION_3	OPTION_4
003	3	2	3
004	9	3	9
006	9	6	6
011	5	3	5
012	4	2	3
013	3	2	2
014	2	1	1
019	3	2	3
021	1	0	0
022	3	1	2
025	1	0	0
028	2	1	2
033	1	0	0
035	1	0	0
037	11	10	10
038	2	0	0
044	2	1	1

This result raises the question about the nature of the geography of the points in relation to the census tract boundaries in these places and how it may impact subsequent linkage with area-based correlates. In sum, results are twofold: 1) a spatial pattern of child pedestrian injury incidents is often aligned with census tract boundaries, and 2) different spatial join procedures produce differential assignment of points to the polygons in which they are located.

## Discussion

### Observations and implications

This study produces two important findings to which researchers should be alerted in the near term. The first is both conceptual and methodological and focuses on the implications of a coupled point-boundary geographic pattern of child pedestrian injury incidents. Near Analysis revealed that, in this case, 46 out of 100 incidents occurred within 30m of a census tract boundary. In effect, this means, that if the incident occurs in one lane of the road, it is assigned to that tract, but take a few steps further into the adjacent lane, and now the incident is assigned to that tract instead. Such a small distance may result in large differences in the number of incidents counted as occurring in each tract and being associated with a different set of socioeconomic contextual variables.

**Fig 6:**
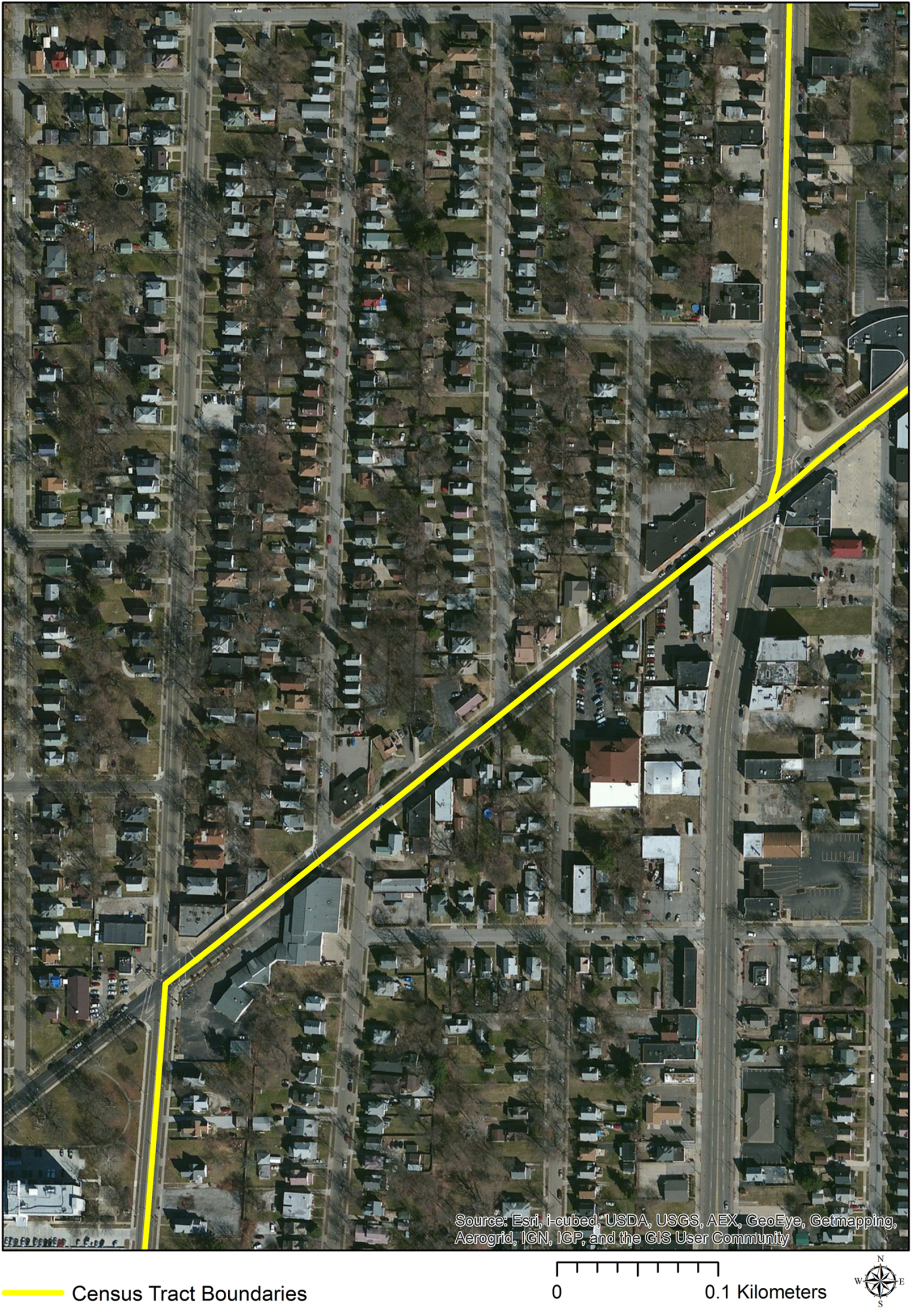
Example of a census tract boundary in real-world context.

Roads are often used as census tract boundaries as they are relatively stable, visible, and identifiable features [25]. Roads are also the common location of pedestrian injuries. Therefore, while using census tracts to study the area-based correlates of many health outcomes, even of injury types, this geographic unit presents a special problem in the case of pedestrian injuries as they are often occurring at the boundaries between tracts. Conceptually, when so many of this type of injury occur at tract boundaries, is it valid to assign them only to the tract in which they occur to investigate area-based correlates? Perhaps using the census data in which the incident “falls within” is only partially satisfactory. Unfortunately, despite the many advances enabled through GIS, it is limited in that polygon boundaries are necessarily represented as firm lines, with one set of values assigned to one side and another set of values assigned to the other (Fig 6). However, the reality is that it is unreasonable to expect that human spatial patterns and processes respect a hard stop at a census tract boundary. It is more realistic to expect that these boundaries have a fuzzy characteristic that is not easily represented in a GIS environment.

The second issue of immediate concern, which is technical in nature, is how are points assigned to polygons in GIS when they are located on a boundary? Option 1, the standard and most intuitive approach, resulted in multiple counting of each border incident which propagated into a larger number of incidents in the tracts than what was reported in the original data set. This led to investigation of alternative approaches. In Option 3, each point was appended with the attributes of the polygon that it falls inside. This meant that every point representing a child pedestrian injury incident was assigned the name of the census tract in which it was located, as well as any other variables included in the tract data. However, this study demonstrated that those points on the border where not assigned to a tract. By summarizing the newly appended tract field, a table was created with counts of incidents for each tract in which they occurred. This table was then joined to the census tract layer to create the choropleth map and conduct the same analysis as would result from Option 1. Option 4 enabled the same output, but also included a field calculating the distance from the points to their nearest polygon boundary and when the point was located inside the polygon, the distance was zero. This option assigned all points to only one tract each. These results should provide guidance to the numerous studies aggregating health outcomes to the census tracts in which they occur, especially those investigating outcomes that may be geographically associated with census boundaries. Pedestrian injury in general, and child pedestrian injury in particular, is one area where the growing body of research [13–16, 21, 25, 26–28] is becoming aware of inherently geographic issues within their studies–those identified through this investigation should be added to the list.

### Identifying and managing uncertainty

The findings of this study demonstrate that using the standard spatial join approach in a GIS, points located on a polygon boundary are counted as falling inside all adjacent polygons. This occurs without any alert to the user, leaving the research to proceed without recognizing that a) the total number of points has increased from the original dataset and b)individual counts of points in particular polygons where a number of points are located on the boundary may be inflated above the expected value. This source of uncertainty is possible in any spatial join procedure of points to polygons, but especially those where the spatial distribution of points is naturally aligned with areal unit boundaries. Therefore, practical approaches are needed immediately for identifying and systematically managing this uncertainty. Table 3 offers a three-stage protocol to address this issue.

**Table 3:** Protocol for identifying and managing point-in-polygon aggregation uncertainty.

Stage	Procedures
1) Observation	Overlay point data (e.g., incidents) with polygon data to which they will be aggregated (e.g., census tracts). Observe the distribution of points in relation to polygons–are they visibly within the polygons, or do they appear to also intersect with boundaries? If visual observation alone can confirm absence of intersection, then the study may proceed without using additional stages in the protocol. If this cannot be confirmed, then the next stage of analysis should be performed.
2) Analysis	a) Conduct Near Analysis to calculate distances between points. If results confirm the absence of intersection, then the study may proceed without using additional stages in the protocol. If this cannot be confirmed, then secondary and/or tertiary analyses are required. b) If points intersect polygons boundaries (NEAR_DIST = 0), then query the attribute table with NEAR_DIST calculations to identify the points intersecting the boundaries. This will identify where uncertainty will arise in the aggregation process. If working with a large dataset, it may also be useful to look for clustering in the data where this concern may be more prominent. c) Conduct Kernel Density Estimation (KDE) to visualize hotspots of potential uncertainty; use of spatial statistics such as Local Moran’s I or GI* may be used to further quantify spatial autocorrelation in these areas of concern.
3) Management	If intersecting points are numerous and widespread such that they cannot be studied and then assigned to an appropriate polygon on a case-by case basis, then use the polygon-in-point (Option 4) spatial join approach to ensure that these points are not counted multiple times.

In sum, these recommendations are easy and quick first steps to integrate into existing GIS data protocols when needing to aggregate point data into the polygons in which they are located. They are simple steps to identify and manage uncertainty, as well as to provide transparency in interpreting results in the numerous studies where GIS is used to understand the relationship between health and place. In this case, GIS should be a tool that provides greater insight into the socioeconomic contexts that result in child pedestrian injuries. However, as they are often spatially distributed along roads that serve as census tract boundaries, unless protocols are adjusted to account for boundary effects on aggregation, it could be muddying the waters.

### Looking beyond big cities

This investigation was initiated through a process that began with observation of points overlaying census tracts. Despite the simplistic nature of this exercise, observation remains essential. In this case, GIS facilitated identification, visualization and then quantification of this geographic pattern. In part, this positive outcome is due to the relatively small size of the study area and low number of incidents which stands in contrast to existing research, most of which has been conducted in large urban areas [13–14, 21, 26]. In major metropolitan areas, the census tracts are smaller and there are usually more points on the map. The result of these large study areas with small tracts and many incidents is difficulty in observing macro-level spatial patterns. Looking at the data in a GIS provides a snapshot of a sea of points, with little ability to see what lies beneath them. It would be unusual to clearly see a boundary relationship between points of incidents and census tracts in these types of study sites when looking at the area in its entirety. In this case, working in a mid-sized city is a benefit as it has sufficient numbers of incidents to cause concern and require analysis, but the numbers and geographic area of their distribution are not so large as to preclude quick, clear visualization. Looking beyond big cities to the mid-sized and smaller towns results in less data distributed over less space, which can lead to greater clarity in observation of geographic patterns.

### Limitations

The contributions of this study and their implications for current and future research must be considered in the context of the project’s limitations. First, the results are only for one mid-sized U.S. city using police incident data over a three-year period. Additional investigation is needed in smaller and in larger areas with different numbers of incidents and geographies of census tracts (e.g., urban areas where tracts are smaller and more numerous and rural areas where they are larger and fewer). Comparative studies across such types of sites will enable understanding of what geographic conditions create a coupling of numerous pedestrian injury incidents with census tract boundaries. Furthermore, this study did not examine the degree of uncertainty that these discrepancies in counts present to subsequent analysis of child pedestrian injury with area-based correlates. For example, how much do census data vary from tract to contiguous tract? Further investigation must be conducted to identify if and how much this issue affects results. Finally, the findings on spatial join approaches and their discrepant results presented in this study are only for a specific GIS software and version. Although the software utilized in this project is the most common, there is growing adoption of other packages and these questions should be investigated in them as well. These are issues for future research to address.

## Conclusions

To date, there has been no reason to question the standard GIS practice of aggregating points to polygons. However, this study reveals that investigating this process and its implications for reported relationships between area-based correlates and health outcomes is needed. This is a particularly pressing concern for research on phenomena that may be tightly coupled with census boundaries.

This study highlights not only a technical concern, but also a more substantive one about the geography of phenomena and their core-periphery relationships to administrative boundaries. Analysis of census tract socioeconomic data has offered valuable understanding of the varied and complex linkage between health and place. Though most health outcomes and processes are not spatially tied to roads and other features of the environment that serve as tract boundaries, some necessarily are linked in this way. Research is needed to identify potentially boundary-dependent processes and outcomes.

With the rapid expansion of GIS throughout public health and the growing acknowledgment of the powerful insights this technology can offer, this study offers a caution. Scholars should pause to critically reflect about the often “black box” nature of the technology and our own personal limits in understanding its operations. Developing formal protocols for use of GIS techniques (even the most basic ones) in public health can improve transparency and confidence as this line of inquiry continues apace to yield real-world benefits for people’s health.

## Supporting information

S1 File PLOS_One_Minimal_Dataset.xlsx. The records listed in this file can be accessed online: http://online.akronohio.gov/apdonline/reportlookup/EULA.aspx?referrer=ReportLookup.
(XLSX)
